# The Definition, Diagnosis, and Management of Giant Splenic Artery Aneurysms and Pseudoaneurysms: A Systematic Review

**DOI:** 10.3390/jcm13195793

**Published:** 2024-09-28

**Authors:** Valerio Rinaldi, Giulio Illuminati, Roberto Caronna, Giampaolo Prezioso, Piergaspare Palumbo, Paolina Saullo, Vito D’Andrea, Priscilla Nardi

**Affiliations:** Department of Surgery, Sapienza University of Rome, Viale Regina Elena 324, 00161 Rome, Italy; giulio.illuminati@uniroma1.it (G.I.); roberto.caronna@uniroma1.it (R.C.); giampaolo.prezioso@libero.it (G.P.); piergaspare.palumbo@uniroma1.it (P.P.); paolina.saullo@uniroma1.it (P.S.); vito.dandrea@uniroma1.it (V.D.)

**Keywords:** splenic artery aneurysm, splenic artery pseudoaneurysm, giant splenic artery aneurysm, giant splenic artery pseudoaneurysm, huge splenic artery aneurysm, huge splenic artery pseudoaneurysms

## Abstract

**Background/Objectives**: Giant splenic artery aneurysms (SAAs) and pseudoaneurysms (SAPs) represent rare conditions, characterized by a diameter greater than or equal to 5 cm. The risk of rupture is increased compared to common SAAs and SAPs, necessitating urgent treatments to prevent it. **Methods**: This systematic review was conducted through a comprehensive search involving the PubMed, Google Scholar, and Scopus databases. A total of 82 patients and 65 articles were included in the analysis. For each patient, we investigated age, sex, symptoms, comorbidities, the presence of a true or a false aneurysm, the dimensional criteria used to define dilations as giant aneurysms or pseudoaneurysms, the dimension of the two greatest diameters, imaging studies, surgical treatment, post-operative length of stay (LOS), and post-operative follow-up. **Results**: The results revealed a similar incidence in both genders (43 males vs. 39 females) with a median age of 55.79 years. The most frequently described symptom was pain (59.76%). Thirteen cases were false aneurysms and 69 were true aneurysms. The mean greatest diameter was 9.90 cm. The CT scan was the most utilized imaging study (80.49%). Open, endovascular, and hybrid surgery were performed in 47, 26, and 9 patients, respectively, with complication rates of 14.89%, 23.08%, and 22.22% occurring for each treatment. The post-operative LOS was 12.29 days, 2.36 days, and 5 days, respectively. The median follow-up was 17.28 months overall. No recanalization was observed after endovascular procedures during the follow-up period. **Conclusions**: The dimensional criterion to define SAAs and SAPs as giant was most frequently that at least one diameter was ≥ 5 cm. The CT scan was the most frequently utilized radiological study to diagnose giant SAAs and SAPs. Finally, endovascular procedures, open surgeries, and hybrid treatments presented similar post-operative complication rates. The post-operative LOS was lower for the endovascular group, and the follow-up period did not show aneurysm recanalization in any patients.

## 1. Introduction

Visceral aneurysms constitute a significant concern in vascular pathology. The risk of rupture exposes patients to eventual hemorrhagic shock and death. Aneurysms represent a saccular or fusiform dilation of the involved artery or vein, exceeding 50% of the normal diameter of the vessel. If the dilation is less than 50% of the standard diameter, the condition is described as ectasia [1]. Abdominal aneurysms represent a critical pathological condition due to the risk of intraperitoneal or retroperitoneal rupture, gastrointestinal fistula, or arteriovenous fistula, all of which can result in hemoperitoneum, hemorrhagic shock or cardiogenic shock. Additionally, the compression of the surrounding organs poses a relevant risk. The most frequent type of abdominal aneurysm is represented by infrarenal abdominal aorta aneurysms, followed by iliac artery aneurysms. Splenic artery aneurysms (SAAs) are the most frequent type of aneurysm arising from visceral arteries among the general population [2]. SAAs are associated with a significant risk of rupture and hemorrhagic shock, particularly if the aneurysm reaches considerable dimensions. Based on dimensional and anatomical criteria, a surgical, endovascular, laparoscopic or hybrid treatment may be indicated. In some cases, a “watch-and-wait” approach is preferred, depending on the patient’s overall health and comorbidities. Aneurysmal dilation must be differentiated between true aneurysms and false aneurysms (pseudoaneurysm). This distinction is based on histological findings revealed by microscopic evaluation: If the arterial layers involved in the vascular dilation are preserved, it is defined as a true aneurysm. Conversely, if the structural layers are subverted, fused, and replaced by fibrotic processes, the aneurysm is called false or a pseudoaneurysm. Radiological imaging could be useful to evaluate if the affected arterial segment is altered by a true or false aneurysm [3]. The surgical treatment for common SAAs or splenic artery pseudoaneurysms (SAPs) is a relevant procedure, particularly if the aneurysm’s dimensions reach a considerable diameter. Many studies in the medical literature define SAAs or SAPs as “giant” when their diameters reach 5 cm [4]. Others refer to giant SAAs or SAPs when the principal diameter is at least 10 cm [5]. Other systematic reviews have been already published in the medical literature; however, different inclusion and exclusion criteria have been utilized [3,4,5,6]. The aim of our study is to determine, through a comprehensive systematic review, which is the most frequent definition of giant SAAs or SAPs, according to the dimensional criteria most commonly adopted in the medical literature. We aim to define the epidemiological, etiological, clinical, and dimensional characteristics of giant SAAs and SAPs. Additionally, we report the principal diagnostic tools currently used to diagnose SAAs or SAPs, the frequency of traditional, laparoscopic, hybrid, and endovascular treatments for giant aneurysms and the short- and long-term outcomes of these procedures, described in the current literature.

## 2. Materials and Methods

**Figure 1:**
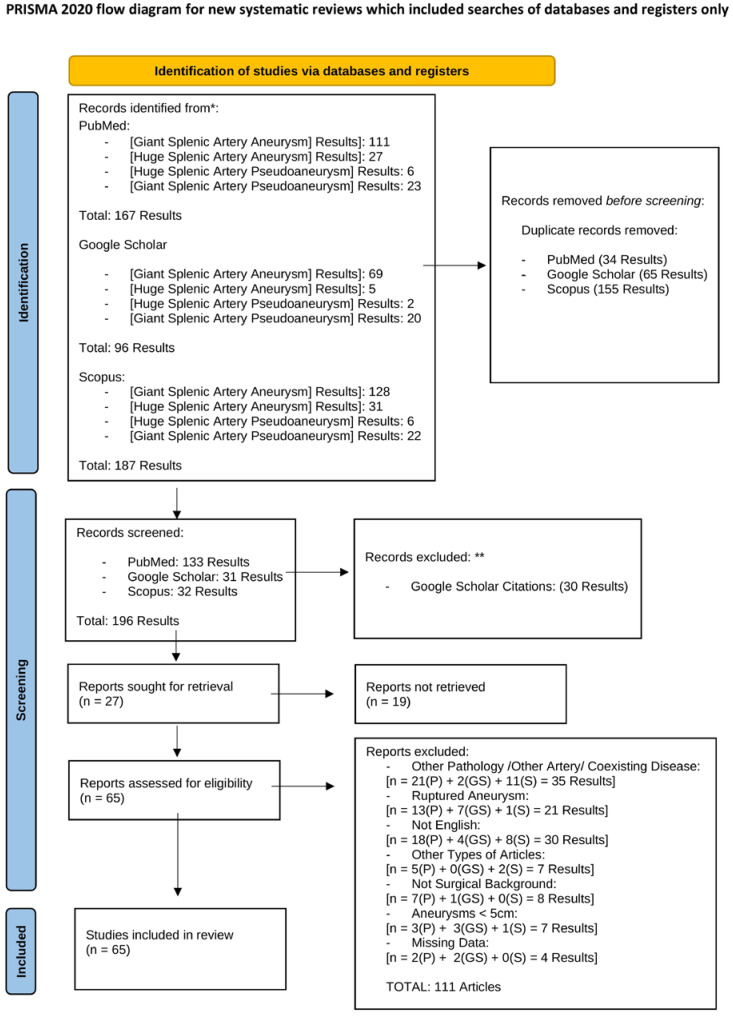
The PRISMA statement. * Consider, if feasible to do so, reporting the number of records identified from each database or register searched (rather than the total number across all databases/registers). ** If automation tools were used, indicate how many records were excluded by a human and how many were excluded by automation tools.

The article search started on 1 March 2024 and continued until 8 September 2024. The literature review involved the PubMed, Google Scholar, and Scopus databases. For each database, we conducted four distinct searches, using the following keywords: “Giant Splenic Artery Aneurysm”, “Giant Splenic Artery Pseudoaneurysm”, “Huge Splenic Artery Aneurysm”, and “Huge Splenic Artery Pseudoaneurysm”. The search was conducted according to the PRISMA statement (Figure 1).

Articles presented in languages other than English and results without available full-text versions were excluded. The inclusion criteria were the following: articles written as case reports or case series with a surgical focus and setting, and patients affected by aneurysms or pseudoaneurysms ≥ 5 cm in at least one of the reported diameters (for each aneurysm, a maximum of two diameters were collected). When three diameters were described, the first and second by size were selected. If only one diameter was available, it was recorded and included. Furthermore, we included only hemodynamically stable patients, without radiological signs of aneurysm rupture, because these conditions could have been responsible for the prolongation of the average post-operative length of stay (LOS). The exclusion criteria were the following: articles not in English; not a case report or case series article; patients not treated surgically; aneurysm dimension < 5 cm; missing data articles; and conditions of hemodynamic instability and aneurysm rupture (including contained ruptures). We also excluded aneurysms arising from other visceral arteries (renal arteries, hepatic artery, celiac trunk, and gastro-duodenal artery), or articles related to other clinical topics, such as articles characterized solely by a radiological focus. Additionally, articles describing patients with other significant or acute diseases that would have necessitated a different and more complex surgical treatment were excluded.

The PubMed search using the keyword “Giant Splenic Artery Aneurysm” produced 111 results. Fifty-five articles were excluded because they were not written in English; were not case series or case reports; did not highlight any surgical background; reported on hemorrhagic shock or aneurysm rupture; reported on concomitant diseases that could have been accountable for severe complications during the hospital stay; described the involvement of other pathologies or other arteries; reported aneurysms with diameters < 5 cm; were unretrieved articles; or were articles with missing data. Starting from this initial search, we enrolled 56 articles describing patients affected by SAA or SAP, all with dimensions greater than 5 cm in at least one diameter, and treated electively. Three articles reported 3 different patients affected by multiple giant splenic artery aneurysms. Only the largest aneurysm was considered for each patient. The PubMed search using the keyword “Huge Splenic Artery Aneurysm” produced 27 results. A total of 3 papers were definitively included, excluding the already chosen articles (7) and the unsuitable ones (17). The search using the keywords “Giant Splenic Artery Pseudoaneurysm” and “Huge Splenic Artery Pseudoaneurysm” on PubMed led to 23 and 6 results, respectively. No articles from this search were included in the final database. The Google Scholar search using the keyword “Giant Splenic Artery Aneurysm” revealed 69 results and 5 articles. The remaining works were either already enrolled or inconsistent with our inclusion criteria. The search with the “Giant Splenic Artery Pseudoaneurysm”, “Huge Splenic Artery Aneurysm”, and “Huge Splenic Artery Pseudoaneurysm” keywords yielded 20, 5, and 2 results, respectively. Only 2 articles were selected from these searches. The SCOPUS search using the keyword “Giant Splenic Artery Aneurysm” led to 128 results, from which only 1 new article was enrolled. The search for the “Giant Splenic Artery Pseudoaneurysm”, “Huge Splenic Artery Aneurysm”, and “Huge Splenic Artery Pseudoaneurysm” keywords produced 22, 6, and 31 results, resulting in 1 new article. Finally, we included 65 articles encompassing 82 patients (Table S1, attached file). For each eligible patient, we gathered data about sex and age, clinical symptoms and signs, isolated comorbidities, and any underlying conditions that might have contributed to the development of SAAs or SAPs. We also distinguished between true and false aneurysms based on histological/radiological findings. We examined whether the enrolled articles described the dimensional criteria used to classify an aneurysm as “giant”. We recorded the precise dimensions of the first and second diameter, if both were provided. If three diameters were reported, the largest diameter was considered the “maximum” and the second-largest diameter was considered the “minimum” in terms of dimensions. The smallest of the three diameters was not considered because it was rarely described in all the involved articles. Moreover, we investigated the radiological and diagnostical tools used pre-operatively for each patient. The most commonly employed tools included the EcoColorDoppler Ultrasound, Contrast Enhanced CT scan or CT Angiography, Contrast Enhanced Magnetic Resonance Imaging (MRI), and selective angiography. We recorded which tool provided the precise diameter size for each aneurysm, if described. Occasionally, different imaging modalities yielded different dimensions, which could also differ from post-operative macroscopic and radiological evaluations. In our systematic review, preference was given to CT scan evaluations for determining aneurysm dimensions, when available. We documented the specific type of surgical treatment performed for each patient, categorizing procedures into endovascular techniques, open laparotomy, or laparoscopic surgeries, as well as hybrid approaches combining endovascular and open or laparoscopic methods. We also searched for complications, including instances of aneurysm recanalization following endovascular exclusion, and documented the length of post-operative hospitalization in days, along with the duration of post-operative follow-up periods. All articles were assessed based on the CARE Guidelines Checklist [7]. Two articles met 5 out of the 13 criteria of the CARE Guidelines. Additionally, 3, 8, 11, 26, 24, and 9 articles met 6, 7, 8, 9, 10, and 11 criteria, respectively, of the CARE Guidelines Checklist. Finally, we compiled all the collected patients’ parameters into two tables for comprehensive reporting: Tables S1 and S2 (attached files). Table S1 shows the data collected for every patient through the various articles. Table S2 report a summary of all data evaluated.

The principal limitations of our study are represented by the relative heterogeneity in the definition of giant splenic artery aneurysms and pseudoaneurysms in the included studies. Furthermore, our revision lacks a pre-registered protocol. All the results and the mean values were observed and reported after the evaluation of every single article.

Ethical approval was waived by the local Ethics Committee of University “La Sapienza” of Rome in view of the nature of the study (retrospective review process).

## 3. Results

Our research included 57 case reports (87.69%) and eight case series (12.31%), involving a total of 82 patients. For patients with multiple splenic artery aneurysms (SAAs) or pseudoaneurysms (SAPs), only the largest was included.

### 3.1. Epidemiology

Out of the 82 patients, 39 were females (47.56%) and 43 were males (52.44%). Their ages ranged from 22 to 87 years with a median age of 55.79 years. The median age was higher among males (59.58 years) compared to females (51.65 years).

### 3.2. Symptoms and Signs

The primary symptom reported was pain, observed in 49 patients (59.76%), predominantly localized in the left upper quadrant of the abdomen (20 out of 49, 40.82%). Pain occasionally radiated to the back, epigastrium, or left flank. Multiple signs and symptoms were often described in a single patient. A palpable mass was noted in 23 patients (28.05%), often associated with abdominal pain and characterized by a pulsatile presentation in 20 patients (86.96%). Fourteen patients were asymptomatic (17.07%). Other symptoms included upper gastrointestinal bleeding, anemia, nausea, vomiting, jaundice, and compression-related symptoms such as ascites and splenomegaly, noted in 11 patients (13.41%). Extrahepatic portal vein obstruction (EHPVO) was found in four patients (4.88%), and fever was commonly reported. An arteriovenous fistula between the aneurysm and the surrounding veins (portal vein and splenic vein) was described in three patients (3.66%). Our study included 13 cases of SAPs (15.85%) and 69 cases of SAAs (84.15%).

### 3.3. Comorbidities

Several pathologies potentially contributing to aneurysm development were identified, including cardiovascular diseases such as systemic arterial hypertension, dyslipidemia, and type 2 diabetes mellitus. Many patients were smokers or former smokers. Others presented a significant history of pancreatic diseases, including chronic and recurrent pancreatitis or pancreatic pseudocysts that were noted in 15.85% of patients, often associated with alcohol consumption. Two patients reported significant abdominal trauma in the past, with no other relevant pathologies identified. One patient was pregnant at the time of diagnosis, and another one had given birth shortly before the diagnosis. For 31 patients (37.80%), no concomitant diseases or comorbidities were described.

### 3.4. Dimensional Criteria Definition

Regarding the dimensional criteria used to classify SAAs or SAPs as “giant”, only 24 articles (29.72%) provided explicit criteria. Among these, 15 articles (62.50%) defined giant aneurysms as those larger than 5 cm, while 9 articles (37.50%) described giant aneurysms as those greater than 10 cm. Only one paper considered any aneurysm larger than 2.5 cm as giant (4.17%).

### 3.5. Dimensions

The maximum diameter was reported in all included articles, ranging from 30.7 cm to 5 cm, with a mean maximum diameter of 9.90 cm. The second diameter, considered the minimum, was reported for 47 patients (56.63%), including diameters measuring less than 5 cm, with a range from 26.3 cm to 3.6 cm and a mean diameter of 8.85 cm.

### 3.6. Imaging

Pre-operative imaging included CT scans for 66 patients (80.49%). It was used in 51 cases to determine the aneurysm dimension. Gastroscopy was performed in 10 cases (12.20%), 1 of which also involved echoendoscopy. EcoColorDoppler Ultrasound was used in 37 patients (45.12%), providing dimensional data for 5 patients. Selective angiography was conducted in 45 cases (54.88%), including 6 cases where it defined the dimensions of the aneurysms. Magnetic Resonance Imaging was proposed to three patients (3.66%). For 17 patients (20.73%), the specific diagnostic tool used to determine aneurysm size was not specified. In two instances, the aneurysm’s dimensions were assessed post-operatively through macroscopic evaluation. Furthermore, other aneurysmatic dilations were found in 11 patients (13.41%), with 1 patient presenting with a portal vein aneurysm, one aneurysm located in the infra-renal aorta, two aneurysms located in both popliteal arteries of 1 patient, and the other aneurysms located in the splenic artery itself. Overall, five patients presented with two splenic aneurysms, whereas two patients showed multiple splenic aneurysms originating from the splenic artery. In the end, one patient presented two small aneurysms at their post-operative CT scan, probably arising from the hemodynamic variation of the splenic hilum vascularization [8].

### 3.7. Surgical Treatments and Complications

Various surgical approaches were employed, from conventional open surgery, endovascular surgery, and combined hybrid surgery (endovascular + surgical approach), to laparoscopic surgery techniques. Overall, 47 patients (57.32%) underwent open surgical procedures, including distal pancreatectomy and splenectomy (15 cases, 31.91%), splenic artery ligation (1 case, 2.13%), aneurysm flattening and exclusion (6 cases, 12.77%), sometimes with concomitant splenectomy (6 cases, 12.77%), aneurysm excision and splenectomy (11 cases, 23.40%), and aneurysmectomy (5 cases, 10.64%), occasionally with vascular reconstruction (termino-terminal anastomosis) (2 cases, 4.26%). One patient was treated with aneurysmectomy + distal pancreatectomy (2.13%). A laparoscopic approach was proposed for a patient treated with a combined hybrid procedure. Open surgery eventually allowed the excision of surrounding organs comprising the aneurysm itself. For example, Varnavas et al. proposed a concomitant Sleeve Gastrectomy for the erosion and infiltration of the stomach greater curvature involved in the splenic aneurysm [9]. Endovascular procedures are generally not indicated if patients present with complex vascular anatomy. Conversely, they are most frequently preferred if the patients are affected by severe comorbidities that would represent a significant cardiorespiratory and metabolic risk for the conventional open surgery. We reported that 26 patients were treated with endovascular procedures (31.71%): endovascular plug to occlude splenic artery (2 cases, 7.69%); coils e plug (3 cases, 11.54%); embolizing fluid (Lipidol or cyanoacrylate (3 cases, 11.54%); coils (10 cases, 38.46%); stenting (5 cases, 19.23%); coils and stenting (1 cases, 3.85%); coil, plug, and cyanoacrylate (1 case, 3.85%); and thrombin (1 case, 3.85%). Instead, during our research, we found that eight patients were treated with a pre-established hybrid approach (endovascular exclusion of the aneurysm and surgical excision, associated or not to a vascular reconstruction; aneurysm embolization and laparoscopic splenectomy; or coils and thrombin placement followed by aneurysmectomy). It is important to report that another one patient was treated with a hybrid approach because of infectious complications that followed the endovascular procedure. In fact, Mastroroberto et al. proposed a surgical strategy to excise an aneurysm embolized by metal coils that were infected post-operatively [10]. Moreover, Huang et al. proposed a hybrid strategy: an angiographic evaluation associated with a percutaneous injection of a thrombin collagen compound in the aneurysm’s lumen [11]. Connors et al. described an endovascular embolization of the aneurysm and subsequent laparoscopic splenectomy [12]. Overall, combined hybrid treatment was completed in nine patients (10.98%). Regarding surgical complications, we found 14 post-operative adverse events (16.87%). The hybrid surgery procedures reported post-operative complications in two treatments out of nine patients (22.22%): coil infections by *Propinilbacterium acnes* and myocardial infarction. Concerning the endovascular procedures, the following were described: splenic abscess; splenic hypoperfusion; splenic infarction; high amylase levels; cerebellar infarction; and superior mesenteric vein thrombosis due to severe thrombocythemia following thrombin injection inside the aneurysms. The reported post-operative complications involved 6 patients out of 26 treated (23.08%). Open surgical techniques reported several complications (seven patients affected by complications compared to 47 surgical procedures (14.89%), such as pancreatic fistulas; post-operative hemorrhage; and portal vein thrombosis associated with necrotic-hemorrhagic pancreatitis and sepsis, treated with laparotomy, drainage and antibiotics.

### 3.8. Length of Stay and Follow-Up

The mean LOS period was 8.79 days for the 39 cases in which LOS was reported. Considering the 25 patients treated surgically in which post-operative LOS was described, the mean period was 12.29 days. The mean LOS period described after endovascular treatment was reported in 11 patients (2.36 days), considering exclusively the first hospitalization and excluding re-admissions for complications. For example, Shetty et al. [13] described a surgical case in which their patient was readmitted twice after discharge for a total LOS of 3 + 2 + 5 days. Including the readmissions, the mean LOS for all patients would have been 3 days. For the hybrid treatment, only three papers reported the post-operative LOS that reached a mean period of 5 days. Mastroroberto et al. reported a significant complication (coil infection) that led to a re-admission, resulting in the lengthening of the mean LOS period to 14 days [10]. This last patient has been included in hybrid treatment because the reintervention was carried out surgically, while the first treatment was endovascular. We also evaluated the mean follow-up period (17.28 months) for the 39 patients in which it was reported (47.56%). Neither perioperative deaths, nor SAA and SAP recanalization at CT scan evaluation were reported in the selected articles. The mean follow-up period for surgical and endovascular procedures was 13.92 and 20.28 months, respectively. The mean follow-up time for patients treated with a hybrid procedure was 15 months.

The overall results are summarized in Tables S1 and S2 (attached files).

## 4. Discussion

**Epidemiology and definition.** Splenic artery aneurysms (SAAs) represent an arterial dilation exceeding 50% of the normal diameter of the splenic artery. Visceral aneurysms constitute 5% of all intra-abdominal aneurysms and are further classified into renal artery aneurysms and splanchnic artery aneurysms. The category of splanchnic artery aneurysms includes both true aneurysms and false aneurysms (pseudoaneurysms). Splenic artery aneurysms account for approximately 60% of all splanchnic artery aneurysms, followed by hepatic artery aneurysms, superior mesenteric artery aneurysms, celiac trunk aneurysms, gastric and gastroepiploic artery aneurysms, and gastroduodenal artery aneurysms. Splenic artery aneurysms rank third in frequency among intra-abdominal aneurysms, after abdominal aortic aneurysms and iliac artery aneurysms. Autopsy studies and angiographic investigations have reported incidences of SAAs in the general population as 0.09% and 0.78%, respectively [2]. Bedford and Lodge (1960) [14] reported a significant incidence of splenic artery aneurysms (10.4%) in autopsies conducted among a geriatric population (>60 years). Zampieri et al. (2005) [15] suggested that aneurysms larger than 3 cm should be considered rare. According to Trastek et al. (1982) [16], who studied 100 patients with SAAs and SAPs, the risk of rupture is approximately 2–3% for common SAAs and 37–47% for common SAPs. Factors like increased diameter and the third trimester of pregnancy are significant risk factors for aneurysm rupture. Medjoubi et al. (2000) [17] reported a 28% rupture rate in giant SAAs (defined as aneurysms greater than 10 cm in diameter). Long et al. (1993) [18] reported mortality rates of 5–15% following aneurysm ruptures in non-pregnant women. Tijani et al. (2020) [19] described fetal and maternal mortality rates of 95% and 70%, respectively, in pregnant women with SAAs. Other studies have reported mortality rates ranging from 25% to 70% (Atanasijevic et al., 2021) [20]. Al Habbal et al. [21] reported that common splenic artery aneurysms generally showed a male-to-female ratio of 1:4 in terms of incidence. Hamid et al. (2020) [22] highlighted the epidemiological differences between common SAAs and giant SAAs (>5 cm), with giant SAAs showing a male-to-female ratio of approximately 1:1. These findings are consistent with our study, which also includes SAPs. Akbulut and Otan (2015) [3] reported that SAAs were 1.78 times more frequent in males than females, with a median age at diagnosis of 57.5 years for males and 52.7 years for females. Our study describes a similar incidence of giant splenic artery aneurysms and pseudoaneurysms, with rates of 47.56% in females and 52.44% in males, out of a total of 82 patients. The median age at diagnosis for both genders was 55.79 years, ranging from 22 to 87 years. The median age at diagnosis was 51.65 years for females and 59.58 years for males.

**Histology and macroscopic features.** The structural differences between SAAs and SAPs are related to the preservation of the original layers composing the arterial wall. In SAAs, the aneurysmal dilation involves the intima, media, and adventitia, which appear intact, although a progressive degradation of elastic fibers and smooth muscle cells can be observed (Borzelli et al., 2021; Yagmur et al., 2015) [23,24]. In contrast, the pseudoaneurysm’s histology shows the absence of at least one of these structural layers, replaced by fibrotic tissue. Macroscopically, aneurysms present in either saccular or fusiform shapes (Akbulut & Otan, 2015) [3]. Although the majority of papers included in our review define “giant” aneurysms and pseudoaneurysms as those with at least one diameter greater than 5 cm, there is no consensus on the precise definition of giant splenic artery aneurysms. Several studies in our review consider giant aneurysms and pseudoaneurysms as those greater than 10 cm in at least one diameter.

**Etiology.** The etiology and risk factors of SAAs can be attributed to various pathological conditions. Firstly, SAAs and SAPs should be considered as distinct pathologies due to their significant histological differences. Giant SAAs are generally associated with similar risk factors to common SAAs, such as cardiovascular diseases (hypertension, atherosclerosis), smoking, and diabetes mellitus, as well as arteritis, collagen vascular diseases, arterial fibrodysplasia, polyarteritis nodosa, alpha-1 antitrypsin deficiency, and systemic lupus erythematosus (S. Yadav et al., 2012) [25]. Pregnancy is significantly linked to the development of SAAs. Our study observed only two cases of giant SAA in pregnant women; however, it is important to note that SAAs are often associated with a history of multiple pregnancies (Abbas et al., 2002) [26]. Common SAAs appear to be four times more frequent in multiparous women compared to nulliparous women. This phenomenon is likely related to the increased levels of estrogen, progesterone, and relaxin, which induce structural and dynamic changes in the splenic artery wall, including sub-endothelial thickening, fragmentation of the internal elastic lamina, and other histological alterations. Overall, these changes weaken the artery, and a concomitant increase in blood pressure may contribute to aneurysm formation (Sadat et al., 2008) [27]. In conclusion, hormonal variations and changes in splanchnic circulation during pregnancy may contribute to the development, enlargement, or rupture of SAAs, particularly during the third trimester (Akbulut & Otan, 2015) [3]. Portal hypertension is another significant factor that can contribute to increased blood pressure in the splenic artery region, either independently or in conjunction with pregnancy. It is considered both a risk factor for SAA development (Lee et al., 1999) [28] and a symptom associated with giant SAAs. In cases of giant SAAs, there may be an external compression of the portal vein, leading to Extra Hepatic Portal Vein Obstruction (EHPVO) (Aroor, 2014; Shinde, 2021) [29,30]. Orthotopic Liver Transplantation (OLT) (Lee et al., 1999) [28] and liver cirrhosis (Sunagozaka et al., 2006) [31] have also been identified as possible causes of SAA development. In contrast, SAPs are primarily associated with previous surgeries, chronic or acute pancreatitis, pancreatic pseudocysts, and a history of significant blunt trauma or infections (Tessier et al., 2003; Tijani et al., 2020) [19,32]. Chronic and recurrent pancreatitis or pancreatic pseudocysts can lead to anatomical changes and structural remodeling of the splenic artery wall due to their proximity and involvement in inflammatory remodeling processes (Tessier et al., 2003) [32].

**Clinical features.** Several symptoms and clinical signs are closely associated with SAAs and SAPs. Common SAAs are typically diagnosed incidentally during radiological examinations conducted for other clinical purposes. Giant SAPs and SAAs can also be detected incidentally. Our review revealed that despite their larger size, giant aneurysms and pseudoaneurysms were asymptomatic in 14 out of 82 patients (17.07%). Splenic artery giant aneurysms can occur in isolation or in conjunction with other common or giant splenic artery aneurysms (Beksac & Karakoc, 2016; Hamid, Suliman, Spiliopoulos, 2020; Rehman, 2019) [6,33,34]. The primary symptom associated with giant SAAs and SAPs is pain, most commonly located in the left upper quadrant of the abdomen and near the epigastric region (Góes Junior et al., 2012) [35]. Additionally, an abdominal mass may be palpable on clinical examination. Sometimes the mass may be pulsatile or may increase in size in the months leading up to diagnosis. Approximately 60% of our patients presented with abdominal pain and discomfort. Sometimes the pain can be associated with dyspeptic symptoms such as nausea, vomiting, and fullness, which are related to stomach compression caused by the aneurysm itself within the retroperitoneal cavity. Other symptoms such as portal hypertension, splenomegaly, and ascites are linked to the compression of the portal and splenic veins by SAAs (Beksac & Karakoc, 2016) [33]. This pathophysiological mechanism is described as Extrahepatic Portal Vein Obstruction, as demonstrated by Shetty (Shetty et al., 2021) [13]. SAAs and SAPs can also lead to the compression of the extrahepatic bile ducts and jaundice. One of the principal symptoms and signs associated with SAAs and SAPs is aneurysmal rupture and subsequent hemorrhagic shock. Aneurysmal rupture can occur in the peritoneal cavity (the most severe type in terms of hemorrhagic shock and mortality), in the retroperitoneum, or through fistula development into adjacent organs and structures near the aneurysm, such as the colon, stomach, duodenum, and pancreatic Wirsung duct, with associated upper gastrointestinal bleeding (melena, hematemesis, anemia, and hematochezia) (Orsitto et al., 2011) [36]. Furthermore, contact with the portal vein and splenic vein can lead to arteriovenous fistula development, as reported by Agrawal et al. and R. Yadav et al. (Agrawal et al., 2006; R. Yadav et al., 2008) [5,6,7,8,9,10,11,12,13,14,15,16,17,18,19,20,21,22,23,24,25,26,27,28,29,30,31,32,33,34,35,36,37]. Ktenidis et al. described an arteriovenous fistula between the splenic vein and a giant SAA that developed after splenectomy (Ktenidis et al., 2018) [38]. Arteriovenous communications can significantly alter portal circulation, which may be associated with portal vein aneurysms, as described by Khan et al. in their work (Khan et al., 2016) [39].

**Diagnosis.** Diagnostic tools utilized for detecting SAAs and SAPs, such as the Contrast Enhanced CT scan, CT Angiography, EcoColorDoppler Ultrasound (ECD US), and Contrast Enhanced Magnetic Resonance Imaging (MRI), have become indispensable in perioperative management. CT scan is considered the most suitable tool for evaluating SAAs, particularly for differentiating them from pancreatic neoplasms, enlarged lymph nodes, post-surgical collections, and lymphoma localization. However, the CT scan is not recommended for pregnant patients, for whom MRI is preferred to avoid exposure to X-rays, offering comparable results in terms of specificity and sensitivity. ECD US serves as the first-line diagnostic tool for evaluating clinical suspicion of SAA. Additionally, it is employed as a complementary examination to assess potential fistulas and/or arteriovenous shunts. ECD US is a feasible, cost-effective, and easily performed examination, although it is inherently operator dependent. Angiography is considered as the gold standard tool, serving both diagnostic and therapeutic purposes due to its capability for endovascular treatment. However, it carries a significant risk of complications during or after percutaneous arterial access and angiographic procedures (Kayacetin et al., 2006) [40].

**Treatment.** The management of splenic artery aneurysms (SAAs) and pseudoaneurysms (SAPs) has been critically reviewed due to the significant risk of rupture associated with these pathologies. According to guidelines from the Society for Vascular Surgery [40], non-ruptured SAPs of any size should be treated due to their rupture risk. SAAs should be treated in women of childbearing age, in cases where they are larger than 3 cm in diameter, in cases where they demonstrate an increase in size, or in symptomatic patients with acceptable surgical risk. Observation is preferred for small SAAs (<3 cm) in patients with significant comorbidities. Endovascular treatment is generally recommended as the first-line approach, while surgical methods are preferred for aneurysms located around the splenic hilum (Chaer et al., 2020) [41]. Other studies suggest that aneurysms with a maximum diameter of 2 cm should be treated in pregnant or fertile patients with portal hypertension or those awaiting liver transplantation (Shabunin et al., 2023) [42]. Strict follow-up is advised for aneurysms or pseudoaneurysms smaller than 2 cm. Endovascular treatment typically results in shorter hospital stays and excellent immediate postoperative outcomes. Various endovascular techniques are utilized for treating giant SAAs and SAPs, including coil embolization, endovascular plugs, stents, Lipidol, or embolizing glue, sometimes in combination. Complications during these procedures may include arteriovenous fistulas at the site of vascular access, pseudoaneurysms, hematomas, infections, and arterial dissection and perforation, necessitating emergency surgical intervention. Local complications, primarily affecting the spleen, such as splenic infarction and abscess formation, may occur due to inadequate residual vascularization of the organ, typically via the remaining short gastric vessels, resulting in altered blood flow (Salimi, Foroutani, et al., 2021) [43]. Long-term complications following endovascular treatment include coil migration, stent displacement and occlusion (Garcarek, 2015) [44], and recanalization of SAAs or SAPs (Chen et al., 2020, Yasumoto et al., 2013) [45,46,47,48,49,50,51,52,53,54,55,56,57,58,59,60,61,62,63,64,65,66,67,68,69,70,71,72,73,74,75,76,77,78,79,80,81,82,83], increasing the risk of rupture, particularly years after treatment when radiological follow-up tends to be less frequent, especially among less compliant patients (Shabunin et al., 2023) [42]. Post-embolization syndrome has also been reported following endovascular treatment for splenic artery aneurysms (Tijani et al., 2020) [19]. Nonetheless, endovascular treatment remains feasible and effective even for giant SAAs and SAPs treated electively, as well as in emergency situations such as hemorrhagic shock due to aneurysmal rupture. Traditional surgical approaches are typically employed for treating vascular diseases like SAAs. Open surgery is commonly preferred for giant SAAs and SAPs, particularly in settings where endovascular techniques are unavailable, whereas laparoscopic surgery is less frequently utilized for giant SAAs and SAPs, except in select cases [84]. Surgical treatment prevents the risk of aneurysm recanalization through the complete excision of the aneurysm itself (Salimi, Foroutani, et al., 2021) [43]. Various surgical procedures are available, with splenectomy combined with distal pancreatectomy being the most commonly utilized approach in our research for giant SAAs and SAPs, particularly due to dense adhesions complicating simple aneurysmectomy. The risks associated with open surgery are hemorrhage, hematomas, infections, damage to surrounding organs, and incisional hernias [85]. Pancreatic fistula is a common complication following splenectomy and distal pancreatectomy, often managed conservatively with perioperative placement of drains (Grade A pancreatic fistula). In cases of significant fistula, radiological drainage (Grade B pancreatic fistula) or reintervention (Grade C pancreatic fistula) may be necessary. Vaccination against encapsulated bacteria is recommended perioperatively when spleen removal is contemplated. Additional surgical strategies include simple aneurysmectomy, aneurysmectomy combined with splenectomy, splenic artery ligation with or without splenectomy, or aneurysmorrhaphy (aneurysm flattening). Termino-terminal arterial reconstruction may be performed when anatomically feasible (Atanasijevic et al., 2021) [20]. Before surgery, it is preferable to consider an aberrant origin of the splenic artery (Illuminati et al., 2007) [46]. Hybrid surgery combines the risks of both endovascular and open surgical procedures but is generally preferred to secure the operative field against arterial ruptures during surgical maneuvers.

## 5. Conclusions

In conclusion, our study focused exclusively on patients diagnosed with giant splenic artery aneurysms (SAAs) or pseudoaneurysms (SAPs) with at least one diameter ≥ 5 cm, yielding several findings. The majority of the reviewed literature defines giant SAAs or SAPs as those having a diameter greater than 5 cm in at least one dimension. SAAs and SAPs were frequently associated with pathologies known to contribute to aneurysm development; however, surprisingly, for a significant proportion of patients (31 out of 82), comorbidities were not described. CT scan evaluation was the most commonly used diagnostic tool to detect giant SAAs or SAPs. Surgical treatment was the predominant approach for managing giant SAAs and SAPs, although endovascular procedures yielded favorable outcomes with lower postoperative complication rates and shorter hospital stays. No instances of giant SAA or SAP recanalization, intraoperative or postoperative deaths were observed during the follow-up period reported. Moreover, hybrid treatment emerged as a potentially valuable alternative for managing giant SAAs and SAPs.

## Data Availability

Enrolled articles can be found in the PubMed, Google Scholar, and Scopus databases as described in the “Materials and Methods” section of the article.

## Supplementary Materials

The following supporting information can be downloaded at: https://www.mdpi.com/article/10.3390/jcm13195793/s1, Table S1: Data collected for every patient of the included articles; Table S2: Summary of all screened data.
